# The Neuroprotective Role of* Origanum syriacum* L. and* Lavandula dentata* L. Essential Oils through Their Effects on AMPA Receptors

**DOI:** 10.1155/2019/5640173

**Published:** 2019-03-11

**Authors:** Mohammad Qneibi, Nidal Jaradat, Mohammed Hawash, Abdel Naser Zaid, Abdel-Razzak Natsheh, Remah Yousef, Qais AbuHasan

**Affiliations:** ^1^Department of Biomedical Sciences, Faculty of Medicine and Health Sciences, An-Najah National University, Nablus, State of Palestine; ^2^Department of Pharmacy, Faculty of Medicine and Health Sciences, An-Najah National University, Nablus, State of Palestine; ^3^Department of Computer Information Systems, Faculty of Engineering and Information Technology, An-Najah National University, Nablus, State of Palestine

## Abstract

*Lavandula dentata* L. and* Origanum syriacum* L. essential oils have numerous health benefits and properties, such as possessing common components with a variant degree of depressive actions in the central nervous system. We investigated the depressive property of these oils on AMPA receptors, which are responsible for most of the fast-excitatory neurotransmission in the CNS and play a critical role in synaptic plasticity. Since excessive activation of AMPARs has been linked to neurotoxicity leading to various pathologies, we hypothesize that these oils have a neuroprotective role by acting directly on the kinetics of AMPARs. Using Gas Chromatography-Mass Spectrometry (GC/MS) and patch-clamp electrophysiology, the essential oils of* L. dentata* flowers and* O. syriacum* leaves were characterized and the whole cell currents were measured with and without the administration of the oils onto HEK293 cells. The current study results showed that the biophysical properties of AMPA receptor subunits showed a decrease in desensitization rate of GluA1 and GluA2 homomers, using* O. syriacum*, while administering* L. dentata* oil decreased the desensitization rate of GluA1 and GluA2 homomers, as well as GluA1/2 heteromers. As for the deactivation rate, both oils slowed the deactivation kinetics of all AMPA receptor subunits. Intriguingly, between the two oils, the effect of desensitization and deactivation was of a greater significance for* L. dentata* oil than* O. syriacum*. Our data suggest that the two oils contain components that are essential to identify, as those active components underlie the oils' neuronal depressive properties reported, and to extract them to synthesize a potent neuroprotective drug to treat neurological diseases potentially.

## 1. Introduction

Many neurological disorders have a chronic pattern and could be very debilitating for the patients. The most common cause of dementia is Alzheimer's disease (AD), which is characterized by progressive neurodegeneration leading to memory loss and cognitive impairment, ending in personality loss, social disinhibition, and death [1]. Epilepsy is a disorder of the synchronized activity of neurons, resulting in a recurrent, unprovoked episode of seizures [2]. Amyotrophic Lateral Sclerosis (ALS) is a fatal neurodegenerative disease that manifests as selective loss of motor neurons in the multiple areas of the nervous system including the brainstem, motor cortex, and spinal cord [3–5]. Moreover, cerebral ischemia, mostly caused by strokes, is one of the most common pathologies that lead to many potentially irreversible neurological deficits [6]. These are a few examples of the many neurological diseases that have all been linked to glutamate toxicity [7].

Glutamate is the primary excitatory neurotransmitter in the mammalian central nervous system (CNS) [8]. Glutamate receptors are classified into metabotropic (mGluRs) and ionotropic (iGluRs) glutamate receptors [9]. The ionotropic receptors are further subclassified into *α*-amino-3-hydroxy-5-methyl-4-isoxazolepropionic acid receptor (AMPAR), N-methyl-D-aspartate receptor (NMDAR), and Kainate receptor, according to their agonists AMPA, NMDA, and Kainate, respectively [10]. AMPAR is responsible for most of the fast-excitatory neurotransmission in the central nervous system, and they critically contribute to the pathology of many neurodegenerative and neuropsychiatric diseases [11, 12]. The function of AMPARs at the synapses relies on the identity of the receptor that is determined by its pore-forming subunits' (GluA1-4) composition. Functional AMPA receptors are assembled into tetramers by one or more distinct subunits and isoforms [13, 14]. In the mature hippocampus, GluA1/GluA2 (GluA1/2) and GluA2/GluA3 (GluA1/3) combinations make the majority of AMPARs [12, 15]. GluA1 is calcium-permeable (without GluA2) and is associated with LTP (long-term potentiation) [16] and LTD (long-term depression) [10], which affect the synaptic plasticity. Due to its abundance in the human brain, its up- and downregulation have been shown to influence the neuronal function in various diseases [17].

The AMPAR subunits that are significant in association with different pathologies are GluA1 and GluA2. GluA1 has essential functions in the synapses including trafficking and insertion of AMPARs. Moreover, in the mature hippocampus, GluA1 is found in the form of GluA1/2 combination, which is impermeable to calcium rather than the GluA1/3 and GluA1 homomeric forms [16]. Its role contrasts that of the calcium-permeable neurotoxic NMDARs, hence its unique role in various nervous disorders [16]. On the other hand, GluA2 is an important subunit as it impacts the biophysical properties of all heteromeric complexes more than all other AMPAR subunits [18]. All GluA2-containing AMPARs do not allow divalent cations to permeate, particularly Ca^2+^ [18]. This is significant, due to the association between the excessive cation influx and the previously mentioned nervous pathologies. Several neurodegenerative and neuropsychiatric diseases have been shown to be associated with excessive activation of AMPA receptors [19, 20]. As a result, pharmacological treatments have long been the first line in therapy for neurological symptoms. However, that type of treatment has modest efficacy and many serious side effects, including Parkinsonism, akathisia, tardive dyskinesia, social withdrawal, or even the risk of cardiac arrhythmias, severe neuroleptic sensitivity reactions, and stroke [21, 22].

Over the years many ligands showing selective competitive inhibitory actions against AMPARs were described [23–25] and efforts to find newer antagonists with enhanced potency, higher specificity, increased water solubility, and longer duration of action continued. To contribute to the evidence-based medicine, the current study investigated essential oils (EOs), which are complex, naturally occurring, volatile compounds synthesized by plants as secondary metabolites, such as French lavender (*Lavandula dentata *L.) and Thyme (*Origanum syriacum*) essential oils.

The* L. dentata* essential oil (LEO) was found to have antimicrobial, carminative antispasmodic, antidepressant, antioxidant, anticholinesterase, and anti-inflammatory effects [26–32]. Oral preparations of LEO exhibit anxiolytic and calming effects with a faster onset of efficacy than first-choice anxiety treatments like serotonin reuptake inhibitors and benzodiazepines [33]. This calming effect is attributed to LEO's inhibitory effect on the autonomous nervous system (ANS) [34]. LEO also has a role in improving cognition, abstract ideas formation, and conceptual understanding of AD patients [35]. Another study demonstrated LEO's ability to improve memory and cognition for AD rats [36]. In addition to its effects on AD, LEO has a positive effect on learning and memory [37], both of which are known to be associated with AMPA glutamate receptors [19, 20]. LEO even showed binding affinity to NMDA glutamate receptors with relevant activity on them [38]. Likewise,* O. syriacum* has many culinary uses and medicinal purposes. Similar to* L. dentata*,* O. syriacum* has antioxidant, antimicrobial, immunomodulatory, anti-inflammatory, and antispasmodic properties [39, 40]. It also has beneficial effects treating several disorders affecting different systems of the body including the cardiovascular, respiratory, and nervous systems [41]. It decreases the level of learning and memory impairment of AD model mice [42]. Finally,* O. syriacum* was recently reported that it partially protects against seizures [43].

Those oils are widely used, and their use is still growing; however, the mechanisms through which they exert their clinical effects are still undiscovered.* L. dentata *and* O. syriacum* essential oils have calming and sedative effects and relevant physiological actions which are of particular interest [26, 27, 33]. Moreover, many studies have demonstrated the safety of using those oils [28, 44, 45].

In this study, the effects of EOs on different homomeric and heteromeric AMPA subunits were tested by compassing effects on the whole cell current as well as the unique biophysical properties of AMPA. Upon biochemical and electrophysiological analysis, the results revealed that* L. dentata* and* O. syriacum* oils distinguishably altered all the studied AMPA subunits' kinetics, both the desensitization and deactivation phases. These results may provide more knowledge on how* L. dentata* and* O. syriacum* oils deploy their considerable activities on the central nervous system and against several neurodegenerative and neuropsychiatric diseases.

## 2. Materials and Methods

### 2.1. L. dentata and O. syriacum Plants Materials

The flowers of* L. dentata* and the leaves of* O. syriacum *were collected from Jenin region of Palestine in April 2018. The studied plants materials were separated from the stems, washed well using water, and dried in the shade at room temperature.

Before that, the taxonomical characterizations were conducted by pharmacognosist Dr. Nidal Jaradat. However, the plants materials were deposited in the Pharmacognosy Laboratory at An-Najah National University and the voucher specimen codes of* L. dentata* and* O. syriacum* plants were Pharm-PCT-1368 and Pharm-PCT-A1729, respectively.

### 2.2. Isolation of the Essential Oils

The essential oils of* L. dentata* and* O. syriacum* were isolated using the ultrasonic-microwave method as described by Jaradat et al. [46] with some modifications. Within the isolation procedure, micro- and ultrasonic waves were used on the suspended plant powder to enhance the extraction process. 1 L round-bottom flask containing 100 g of the dried plants powdered materials with 500 ml water was placed in this apparatus. During the isolation process, the power of the ultrasonic-microwave extractor apparatus was set at 1000 W. The isolation process was carried out at 100°C for 15 min and this procedure was repeated several times for the same plant sample. The obtained EOs were collected into a glass bottle and kept at 2-8°C. The average yields for the isolated essential oils of* L. dentata* and* O. syriacum* were 0.96% and 1.71% v/w, respectively.

### 2.3. GC-MS Study

The chromatograms of the GC-MS were established by utilizing the Shimadzu QP-5000 apparatus. In fact, the GC was equipped with Rtx-5 ms column (0.25 *μ*m thickness, 30 m long, and 0.250 mm internal diameter). The carrier used was helium gas at a standard flow rate of 1 ml per min. The temperature of the injector was programmed at 220°C while the temperature of the oven was adjusted from 50°C (1min hold) at 5°C/min to 130°C and then at 10°C/min to 250°C and then kept isothermally for 15 min. The temperature of the transfer line was set to 290°C. An electron ionization system was used for GC-MS detection, with detector volts of 1.7 KV. A scan speed of 1000 amu/sec and a scan rate of 0.5 s were applied covering a mass range from 38 to 450 M/Z. Using the mass spectrometry data center of the national institute of standards and technology (NIST), the chemical components of the VO were characterized by comparing their MS with the reference spectra in the mass spectrometry data center of NIST; moreover, their Kovats and retention indices were used by comparing them with those in the literature. However, without the use of a correction factor, the quantitative data were electronically obtained from the integrated peaks and area percentages [47].

### 2.4. DNA Preparation

QIAGEN Plasmid Mini Kit was used to prepare up to 20 *μ*g of high-copy plasmid DNA. A selective plate was streaked followed by the selection of a single colony. LB medium was used as a starter culture to be inoculated containing the appropriate selective antibiotic. This was followed by the incubation with vigorous shaking for approximately 8 h at 37°C; 3 ml selective LB medium was then used to dilute the starter culture. The culture was left in the incubator at 37°C for 12–16 h. To harvest the bacterial cells, centrifugation was done followed by the resuspension of the formed pellet with shaking. Subsequently, 0.3 ml of Buffer P2 was added and mixed thoroughly by vigorously inverting the sealed tube 4–6 times. Later, the tubes were centrifuged, and the supernatant was promptly removed as it contains the plasmid DNA.

1 ml Buffer QBT was used to equilibrate a QIAGEN-tip 20, the column could empty by gravity flow, and then the supernatant was applied to the QIAGEN-tip 20 and, by gravity flow, it entered the resin. Buffer QC was used to wash the QIAGEN-tip. This was followed with the elution of the DNA with 0.8 ml buffer QF, and then isopropanol was added to precipitate it. It was mixed and centrifuged immediately then the supernatant was carefully decanted. Ethanol was used to wash the DNA pellet and then centrifuged again, and the supernatant was carefully removed as to not disturb the pellet. Finally, the pellet was air-dried, and the DNA was redissolved in a suitable volume of buffer. Bot spectrophotometry at 260 nm and quantitative analysis on an agarose gel were used to calculate DNA concentration as to determine the yield. A260 readings should lie between the values of 0.1 and 1.0 to judge the reliability of spectrophotometric DNA quantification.

### 2.5. HEK293 Cell Culture and Transfection

Dulbecco Modified Eagle Medium (DMEM) (Sigma, USA) was used to grow Human Embryonic Kidney cells 293 (HEK293). DMEM was supplemented with 10% FBS (fetal bovine serum), 0.1 mg/ml streptomycin, and 1 mM sodium pyruvate (Biological Industries; Beit-Haemek, Israel) at 37°C and 5% CO2 [48]. A twice weekly pass was done until cells reached pass #20. One of two was used for transfection, either jetPRIME transfection reagent (Polyplus: New York, NY) or Lipofectamine 2000 (Invitrogen; San Diego, CA) [49]. For electrophysiology recordings or stereomicroscopy imaging, after transfection, cells were kept for 24 hours and then replated on coverslips coated with Laminin (1 mg/mL; Sigma, Germany).

### 2.6. Electrophysiology

*HEK293 Cell Patch-Clamp Recordings*. HEK293 cells were recorded 36-48 hours after transfection. Recordings were performed at 22°C, at a membrane potential of -60 mV, using the whole cell configuration of the patch-clamp technique via IPA (Integrated Patch Amplifier) (Sutter Instruments, Novato, CA). Membrane currents were digitized using SutterPatch Software v. 1.1.1 (Sutter Instruments) for a short period of time. Sampling frequency was set to 10 kHz, and the low-pass filter was set to 2 kHz. Borosilicate glass was used to fabricate the Patch electrodes with a resistance of 2-4 MΩ. The extracellular solution contained (values are in mM) 150 NaCl, 2.8 KCl, 0.5 MgCl2, 2 CaCl2, and 10 HEPES adjusted to pH 7.4 with NaOH. The pipette solution contains (values are in mM) 110 CsF, 30 CsCl, 4 NaCl, 0.5 CaCl2, 10 Trypsin EDTA solution B (0.25%), EDTA (0.05%), and 10 HEPES, adjusted to pH 7.2 with CsOH. A double barrel glass (theta tube) was used to rapidly apply glutamate and solutions used; the theta tube was mounted on a high-speed piezo solution switcher (Automate Scientific, Berkeley, CA). The open tip potentials were recorded during applying solutions of different ionic strengths after expelling the patch from the electrode to estimate the speed of solution exchange. The 10%–90% solution exchange was typically <500 ms. Data acquisition was analyzed using Igor Pro7 (Wave Metrics, inc) [48]. Borosilicate glass was used to fabricate the Patch electrodes with a low resistance of 2-3 M**Ω**. AMPAR deactivation and desensitization were measured by applying glutamate (10 mM) for 1 ms and 500 ms, respectively. AMPAR-current deactivation and desensitization were fitted with two exponentials and the weighted tau (**τ**_w_) was calculated as **τ**_w_ = (**τ**f x af) + (**τ**s x as), where af and as are the relative amplitudes of the fast (**τ**f) and slow (**τ**s) exponential component.

### 2.7. Statistical Analysis

Significance was compared with AMPAR expressed alone or with AMPAR+EOs (*∗*);* p* value (one-way ANOVA): ^*∗*^*p* < 0.05; ^*∗∗*^*p* < 0.01; ^*∗∗∗*^*p *< 0.001; ns, not significant.

## 3. Results

### 3.1. L. dentata and O. syriacum Essential Oils Phytochemical Components

Tables 1 and 2 depict the GC/MS results of* L. dentata *and* O. syriacum *essential oils components which are obtained from Figures S1 and S2 (found in the supplementary file). The GC/MS results showed the presence of linalyl acetate and linalool to be the major components of* L. dentata *essential oil while thymol and carvacrol were the major components of* O. syriacum *essential oil.

### 3.2. Effect of L. dentata and O. syriacum Oils on the Peak Current of AMPAR Subunits

**Figure 1:**
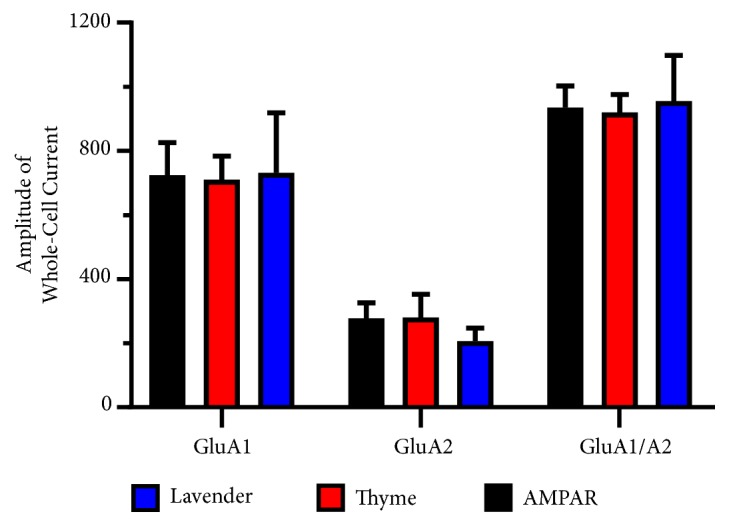
Effect of* L. dentata* and* O. syriacum* oils on the amplitude of the whole-cell current in the absence and presence of both EOs. (A, B, and C) Representative normalized whole-cell current traces of AMPAR recorded upon 500 ms application of 10 mM glutamate to whole-cell recording from HEK293 cells expressing homomeric GluA1 (left), GluA2 (middle), and heteromeric GluA1/2 (right) alone (black) or in combination with* O. syriacum* (red),* L. dentata* (blue).

To inquire direct effects of* L. dentata* and* O. syriacum *oils on AMPAR properties, heterologous expression in HEK293 cells and whole-cell patch-clamp electrophysiological recordings were used to compare them. The amplitude generated by the homomeric GluA1Q, GluA2Q, receptors, and the heteromeric GluA1/A2Q receptor was measured using Integrated Patch Amplifiers (IPA), which enables efficient, low-noise whole-cell recordings. Agonist was applied on AMPAR by using Piezo Fast Exchange solution with 10 mM of glutamate. Data were analyzed using Igor 7 software, as shown in Figure 1.

The effects of* L. dentata* and* O. syriacum *oils on the homomers or heteromeric of AMPA receptors were determined at a fixed concentration of 80 *μ*M and 100 *μ*M, respectively. These concentrations were chosen for their highest effect without affecting the health of the cells. The EOs did not show a meaningful reduction in the average peak current. The peak currents of AMPA receptors subunits in the absence and presence of* L. dentata* and* O. syriacum *oils were 724 ± 101 pA to 732 ± 186 pA or 710 ± 74 pA for GluA1, 935 ± 67 pA to 955 ± 143 pA or 920 ± 56 for GluA2, and 278 ± 48 pA to 207 ± 40 or 280 ± 73 for GluA1/A2, respectively. None of them were statistically significant. The y-axis of Figure 1 is plotted as the amplitude of the whole-cell current in the absence and presence of both Eos vs. the concentration of the* L. dentata* or* O. syriacum *oil in the x-axis.

### 3.3. L. dentata and O. syriacum Oils Alter AMPAR Desensitization in a Subunit-Dependent Manner

**Figure 2:**
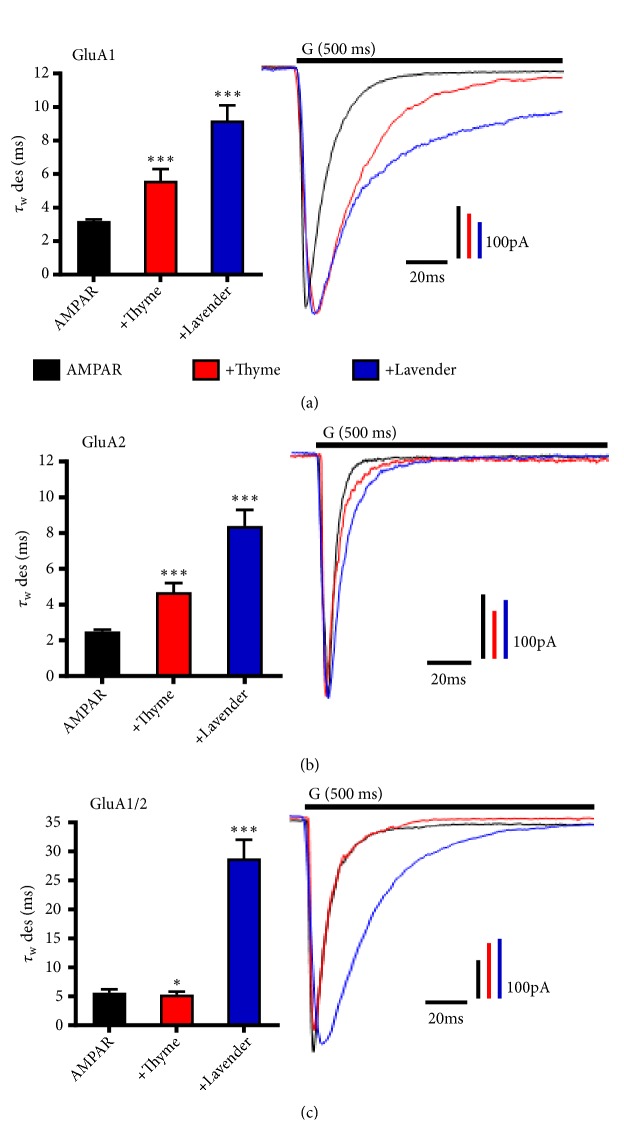
*L. dentata* and* O. syriacum* oils alter AMPAR desensitization in a subunit-dependent manner. ((a), (b), and (c)) Illustrative normalized whole-cell current traces of AMPAR recorded upon 500 ms application of 10 mM glutamate (G, indicated above the current trace) to whole-cell recording from HEK293 cells expressing homomeric GluA1 (top), GluA2 (middle), and heteromeric GluA1/2 (bottom) alone (black) or in combination with* O. syriacum* (red),* L. dentata* (blue). The whole-cell current recording was conducted at −60 mV, pH 7.4, and 22°C. Graphs summarize weighted time constants for desensitization (*τ*_W_ des). Data shown are mean ± SEM; n = 10–20 patches. Significance (one-way ANOVA): ^*∗*^*p* < 0.05; ^*∗∗*^*p* < 0.01; ^*∗∗∗*^*p* < 0.001; ns, not significant.

To elucidate the biophysical effect of EOs by* L. dentata* and* O. syriacum *oils, the effect of each oil on GluA1, GluA2, and the heteromeric GluA1/A2 was characterized and compared with AMPARs alone. Both oils demonstrated an effect on the desensitization of almost all AMPA subunits. Desensitization is caused by the closure of the ion channel pore while the receptors remain in a ligand-bound state [50]. In fact, for* L. dentata* it reduces desensitization of GluA1, GluA2 homomers, and GluA1/2 heteromers (Figure 2). The average desensitization time of GluA1, GluA2, and GluA1/2 was 3.2 ± 0.1 ms, 2.5 ± 0.1ms, and 5.6 ± 0.6 ms, respectively, while in the presence of* L. dentata* it was 9.2 ± 0.9 ms, 8.4 ± 0.9 ms, and 28.8 ± 3.2 ms as shown in Figures 2(a)–2(c). The average rate (*τ*=1/ms) of GluA1 desensitization decreased from 0.31 ms-1 to 0.10 ms-1, ~3.1-fold, GluA2 average desensitization rate decreased from 0.4 ms-1 to 0.11 ms-1, ~3.63-fold, and GluA1/A2 average desensitization rate decreased from 0.17 ms-1 to 0.03 ms-1, ~5.7-fold. Similarly, the* O. syriacum *oil affected GluA1 and GluA2, but not GluA1/2 desensitization time, but it was less when compared to* L. dentata*. The average desensitization time in the presence of* O. syriacum *was 5.6 ± 0.7 ms, 4.7 ± 0.5ms, and 5.3 ± 0.5 ms, respectively, while the average rate decreased ~1.82-, ~1.88-, and ~0.94-fold, respectively.

### 3.4. L. dentata and O. syriacum Oils Modify AMPAR Deactivation in a Subunit-Dependent Manner

**Figure 3:**
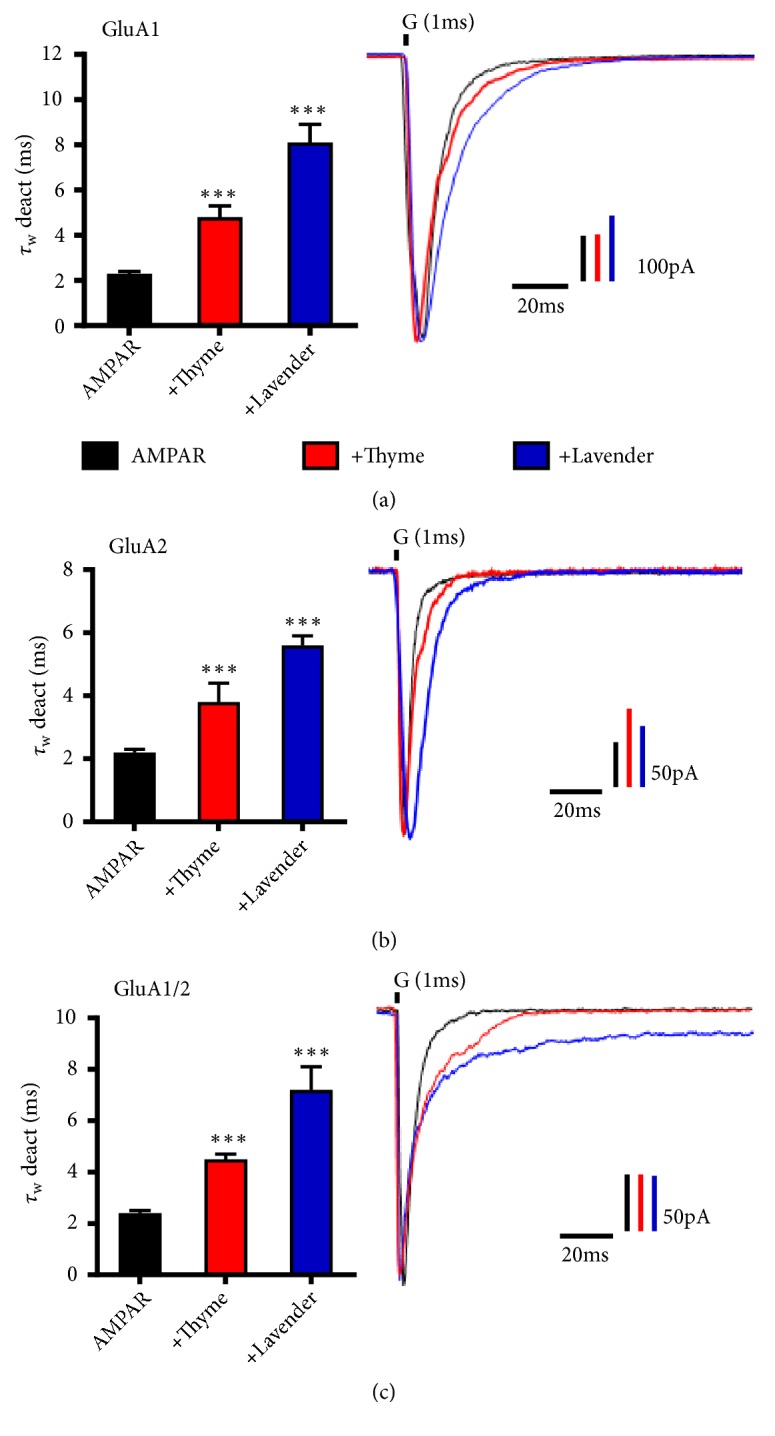
*L. dentata* and* O. syriacum* oils modify AMPAR deactivation in a subunit-dependent manner. ((a), (b), and (c)) Illustrative normalized current responses of AMPAR recorded upon 1 ms application of 10 mM glutamate (G, indicated above the current trace) to whole-cell recording from HEK293 cells expressing homomeric GluA1 (top), GluA2 (middle), and heteromeric GluA1/2 (bottom) alone (black) or in combination with* O. syriacum* (red),* L. dentata* (blue). The whole-cell current recording was conducted at −60 mV, pH 7.4, and 22°C. Graphs summarize weighted time constants for deactivation (*τ*_W_ deact). Data shown are mean ± SEM; n= 10–20 patches. Significance (one-way ANOVA): ^*∗*^*p* < 0.05; ^*∗∗*^*p* < 0.01; ^*∗∗∗*^*p* < 0.001; ns, not significant.

These results motivated the investigation of essential oils on AMPAR deactivation. Deactivation is the characteristic decay in current after a short simulation time following [51]. As shown in Figure 3,* L. dentata* slows deactivation kinetics of GluA1, GluA2 homomers, and GluA1/2 heteromers, but slightly higher than* O. syriacum*. The average deactivation time of GluA1, GluA2, and GluA1/2 was 2.4 ± 0.1 ms, 2.2 ± 0.1 ms, and 2.3 ± 0.1 ms, respectively, while in the presence of* L. dentata* it was 7.2 ± 0.9 ms, 5.6 ± 0.3 ms, and 8.1 ± 0.8 ms as shown in Figures 3(a)–3(c). The average rate of GluA1, GluA2, and GluA1/2 deactivation decreased ~3.2-, ~2.5-, ~3.5-fold, respectively, in the presence of* L. dentata* oil. Furthermore, the* O. syriacum *oil affected GluA1, GluA2, and GluA1/2 deactivation time. Hence, the average deactivation time in the presence of* O. syriacum *was 4.5 ± 0.2 ms, 3.8 ± 0.6 ms, and 4.8 ± 0.5 ms, while the average rate decreased to ~1.9-, ~1.7-, and ~2.1-fold, respectively.

## 4. Discussion

AMPARs have long been described in the pathology of several neurodegenerative and neuropsychiatric diseases, as the overactivation of AMPAR is potently excitotoxic triggering either rapid or delayed neurotoxicity [3, 4]. Such diseases include Alzheimer's disease, ALS, stroke, and epilepsy. In ALS, the activation of AMPA receptors leads to the influx of calcium into neurons in toxic levels, leading to the death of motor neurons; it also has been shown that AMPARs expressed in motor neurons are unusually Ca^2+^ permeable in ALS patients and those patients have high glutamate concentrations [52]. Similarly, in some epilepsies, neuron damage and joint overactivation could happen due to overactivation of AMPA receptors. This manifests as seizure activity in patients affected by those epilepsy types [20]. In ischemia excitotoxicity arises due to oxygen deprivation, which activates voltage-sensitive Ca^2+^ to release high levels of glutamate that will overactivate the neuron [6]. Meanwhile, in Alzheimer's disease, AMPARs disturbed trafficking and reduced synaptic stabilization and regressed synaptic function and cognition [53].

AMPA receptors are ligand-gated ion channels that open a transmembrane ion channel in response to glutamate binding, thus, transforming chemical signals into electrical signals. After the ionotropic receptor has been activated, it undergoes one of two processes: either the ligand unbinds the receptor leading to its deactivation or the ion channel pore closes remaining in a ligand-bound state leading to its desensitization [50]. Namely, deactivation is defined as the natural decay in current after a short simulation time following activation (a fast component of current decay), while desensitization is the natural decay in current after a long simulation time following activation (a slow component of current decay). Both of those biophysical properties can be used as potential targets to treat many neurological disorders.

The behavior of AMPA receptors is complex and many kinetic schemes were used to model such behavior. High-resolution studies conducted on isolated receptor domains and intact receptors further explain the biophysical properties of AMPA receptors, as they demonstrated the ligand binding domains (LBDs) to close as a “clamshell” after the binding to an agonist. When the ligand binds to the clamshell extracellular structure, a conformational change is triggered in the LBD, which pulls apart the linkers connecting the LBD to the ion channel, opening the channel. Subsequently, conformational changes take place in the dimer interface that breaks contacts between LBDs. This change leads to tension relief of the ion channel linkers, allowing the closure of the ion despite the ligand's high-affinity binding [54]. For the receptor to recover from the desensitized state, the ligand must unbind from the LBD, triggering a conformational change that brings the receptor back to the resting state.

The focus of this study is to investigate the homomeric, GluA2, and GluA1 channels in addition to the heteromeric GluA1/A2 channels. The abnormal expression of the unedited isoform of GluA2Q due to editing defect was reported in some diseases. Furthermore, GluA2 is an essential AMPA receptor subunit as it controls the permeability of Ca^2+^ of native AMPA receptors, while the homomeric calcium-permeable GluA1 has essential functions in the synapses including trafficking and insertion of AMPARs, hence their unique role in various nervous system disorders. As for the heteromeric GluA1/A2 channels, it makes up the majority of AMPARs in the mature hippocampus. The time course and amplitude of the little current mediated by AMPA receptor showed that these receptors undergo multiple simultaneous processes including the activation, deactivation, and desensitization of the receptor. The activation of the receptors is the opening of the channel and allows ion influx into the neuron, in response to glutamate binding, thus, transforming chemical signals into electrical signals. After the ionotropic receptor has been activated, it undergoes rapid deactivation or desensitization. Thus, the flow of ions through the channel is terminated by one of those mechanisms.

Essential oils have recently become a source of interest, in response to treating different nervous diseases. The apparent biological effect of EOs on the central nervous system was intriguing to investigate, specifically towards AMPA receptors.* L. dentata* oil has a calming effect which could be partially explained by an inhibitory effect on glutamate receptors [38]. A study showed* L. dentata* to have an affinity to bind to NMDA glutamate receptors and exert inhibitory activity at those receptors, which suggests that* L. dentata* might exercise neuroprotection against the glutamate-induced toxicity either by blocking this glutamate-activated ionotropic receptor or by its calcium channel blocking activity [38].* L. dentata* oil was reported to induce neuroprotection in focal cerebral ischemia [55] and effectively reverse spatial learning deficits in Alzheimer's disease patients with significant improvement in performance [56]. Furthermore, studies on linalool, one of the major components of* L. dentata* oil, demonstrated its ability to inhibit the binding of glutamate in rats [57]. Similarly,* O. syriacum* oil decreases the level of learning and memory impairment of AD model mice [42]. It was recently reported that* O. syriacum* partially protects against seizures [43]. These critical effects of* L. dentata* and* O. syriacum *oils with the established link between AMPA receptors and such neurodegenerative diseases would predict a similar inhibitory pattern on AMPA receptors.

Therefore, the current study investigated the effects of* L. dentata* oil using an electrophysiologic approach. Giving that glutamate is a pharmacological target for the reduction of anxiety and epilepsy, and that its effect is mediated via the AMPA receptor. The effect of* L. dentata* and* O. syriacum *oils was characterized on three different characteristics of AMPA receptors, peak current, desensitization, and deactivation, to detect any modulatory activity.

To investigate the effects on AMPAR gating kinetics in a subunit-dependent manner, first the glutamate-evoked peak current generated by AMPA receptors was measured as well as changes with the application of* L. dentata* and* O. syriacum* oils. The current study did not identify any inhibitory effect of the EOs on the glutamate-evoked peak current generated by AMPA receptors. The results suggest that the EOs did not exert measurable effects on the activation phase time. There was no observed shortening of the activation limb of the AMPA receptor current trace or a statistically significant decrease in the peak amplitude.

Next, the deactivation and desensitization of the receptors were investigated. The deactivation phase represents the fast component of the current decay; thus it is investigated upon rapid application of the agonist and recording the current decay following glutamate removal (1ms). In contrast to the rapid deactivation, the current decay during the sustained application of agonist is how the desensitization, which represents the slow component of the current decay (500ms), was investigated, observing an effect on the kinetics of the desensitization and deactivation of AMPARs.

The results showed an apparent effect of* L. dentata* and* O. syriacum* oils on both the deactivation and desensitization of AMPARs. Upon administration of the oils, the time the receptor spent in the desensitized and deactivated phases was increased, demonstrated by a reduction in the value of tau (1/sec.). Hence, both oils reduced the rate of both kinetics on AMPARs, meaning the duration of these states was prolonged, which indirectly reduces the activity of AMPARs. The postsynaptic signal is accounted by not only the number of AMPARs on the postsynaptic neuron but also, in reality, how many of them are available to bind to an agonist. Thus, by prolonging the duration of desensitization/deactivation of AMPA by decreasing the rate of this kinetics, the risk of neurotoxicity is reduced. This suggests that* L. dentata* and* O. syriacum* oils might affect the AMPA receptor conformation, stabilizing the desensitized/deactivated conformation instead of the resting state.

AMPA receptors mediate synaptic currents at most excitatory synapses, and such currents play a part in the pathophysiology of the discussed disorders. Thus, synaptic depression is needed to counteract these mechanisms, and this could be achieved by altering the mechanisms by which the flow of ions is terminated in the ion channels by glutamate receptors, namely, deactivation and desensitization. The current study sheds light on the potential both* L. dentata* and* O. syriacum* essential oils have in drug synthesis.

## 5. Conclusion

In conclusion, the current study has demonstrated that* L. dentata* and* O. syriacum* essential oils did have a valuable effect on AMPAR kinetics. The effects observed on AMPAR kinetics in these results revealed a decrease in desensitization and deactivation rate, suggesting that* L. dentata* and* O. syriacum* essential oils modify the AMPAR gating kinetics in a subunit-dependent manner. Two different mechanisms for the observed effects of* L. dentata* and* O. syriacum* essential oils on AMPAR kinetics could be proposed, as they might affect AMPAR conformation; first, they might stabilize the desensitized conformation as they slow the kinetics of desensitization. Second, they might similarly stabilize the deactivated form to their effect on desensitization. With the achieved results in this study and the minimal side effects of those oils compared to chemical compounds [33], these oils are very promising for their neuroprotective effects. For future research, all the unique chemical entities found in the* L. dentata *and* O. syriacum* oils would be investigated to establish the most potent compound on AMPARs and see if it can be modified to design a new AMPA-antagonist drug that is effective against specific neurodegenerative diseases. It is apparent that understanding the channel properties of homomeric and heteromeric receptors will generate new possibilities for a mechanism-based inhibitor or drug development to control the function of these channels against neurological diseases.

## Figures and Tables

**Table 1:** *L. dentata* essential oil components.

Essential oils components	R. T	R. I	% of area
L-alpha-Pinene	8.65	R:835	0.1

Santolina triene	9.31	R:746	0.02

Beta-Phellandrene	10.27	R:702	0.005

Sabinene	10.45	R:917	0.1

1-Octen-3-ol	10.69	R:867	0.2

b-Myrcene	11.02	R:795	0.03

3-Octanol	11.4	R:901	0.1

Hexyl ethanoate	12.01	R:911	0.4

2-Carene	12.11	R:764	0.02

1,3,8-p-Menthatriene	12.44	R:825	0.03

P-Menth-8-en-1-olacetate	12.63	R:894	1.3

Eucalyptol	12.74	R:917	1.4

trans-Ocimene	12.96	R:885	0.2

Ocimene	13.39	R:820	0.1

p-Menthadiene	13.84	R:766	0.01

Linalool	15.62	R:921	40.8

*Plinol C*	16.62	R:802	2.6

L-camphor	17.33	R:940	1.8

lsoborneol	17.99	R:745	0.02

Borneol	18.33	R:890	0.5

4-Terpineol	18.46	R:827	0.8

Terpineol	19.22	R:959	4.3

Fenchene	20.3	R:831	0.1

*Linalyl acetate*	21.26	R:940	42.1

(+)-Lavandulolacetate	22.41	R:820	0.4

Carvacrol	23	R:766	0.01

lsobutyl tiglate	23.98	R:705	0.01

alpha-Cubebene	24.59	R:749	0.01

Fenchen	24.96	R:786	0.2

*b-Myrcene*	25.61	R:828	0.4

trans-Caryophyllene	26.99	R:898	1.9

Humulene	28.12	R:782	0.1

Total			100

**Table 2:** *O. syriacum *essential oil components.

Essential oil components	R. T	R. I	% of area
origanene	8.63	R:842	0.1

Camphene	9.29	R:763	0.04

Sabinen	10.44	R:793	0.04

1-Octen-3-ol	10.68	R:828	0.2

Myrcene	11	R:786	0.2

P-Menthadiene	11.64	R:733	0.02

Bilagen	12.1	R:798	0.2

o-Cymene	12.41	R:952	4.2

L-Limonene	12.61	R:777	0.1

Linalyl alcohol	15.54	R:867	8.7

Borneol	18.32	R:893	0.9

4-Terpineol	18.64	R:790	0.8

Alpha-Terpineol	19.21	R:787	0.1

O-Methylthymol	20.82	R:813	0.3

Ca1vacrol	22.76	R:905	16.6

Thymol	23.09	R:896	60.8

Beta-Caryophyllene	26.99	R:917	4.7

Alloaromadendren	27.59	R:811	0.3

Alpha-Caryophyllene	28.12	R:791	0.1

(+)-Ledene	29.27	R:713	0.1

Bergamotol,Z-?-trans-	31.86	R:693	0.1

Caryophyllene oxide	32.01	R:842	1.4

Total			100

## Data Availability

The data analysis data used to support the findings of this study are included within the supplementary information file. Request for access to any additional data should be connected directly to the corresponding author.

## Supplementary Materials

Supplementary Materials See the figures (Figures S1 and S2) in the supplementary folder for the comprehensive GC/MS and whole cell data analysis (see Table S1).
