# Supplementary material for: The Neuroprotective Role of Origanum syriacum L. and Lavandula dentata L. Essential Oils through Their Effects on AMPA Receptors
Source: Biomed Res Int. 2019 Mar 11;2019:5640173. doi: 10.1155/2019/5640173 (PMC6582867; doi:10.1155/2019/5640173)
Supplement: Supplementary Materials — See the figures (Figures S1 and S2) in the supplementary folder for the comprehensive GC/MS and whole cell data analysis (see Table S1). [file 5640173.f1.pdf]

Supplemental Information

The Neuroprotective Role of *Origanum syriacum L.* and *Lavandula dentata L.* Essential Oils through their Effects on AMPA Receptors

Mohammad Qneibi[1,*], Nidal Jaradat[2], Mohammed Hawash[2], Abdel Naser Zaid[2], Abdel- Razzak Natsheh[3], Remah Yousef[1], Qais Abuhasan[1]

SUPPLEMENTAL FIGURES AND LEGENDS

Figure S1. GC/MS chromatogram of *L. dentata* essential oil

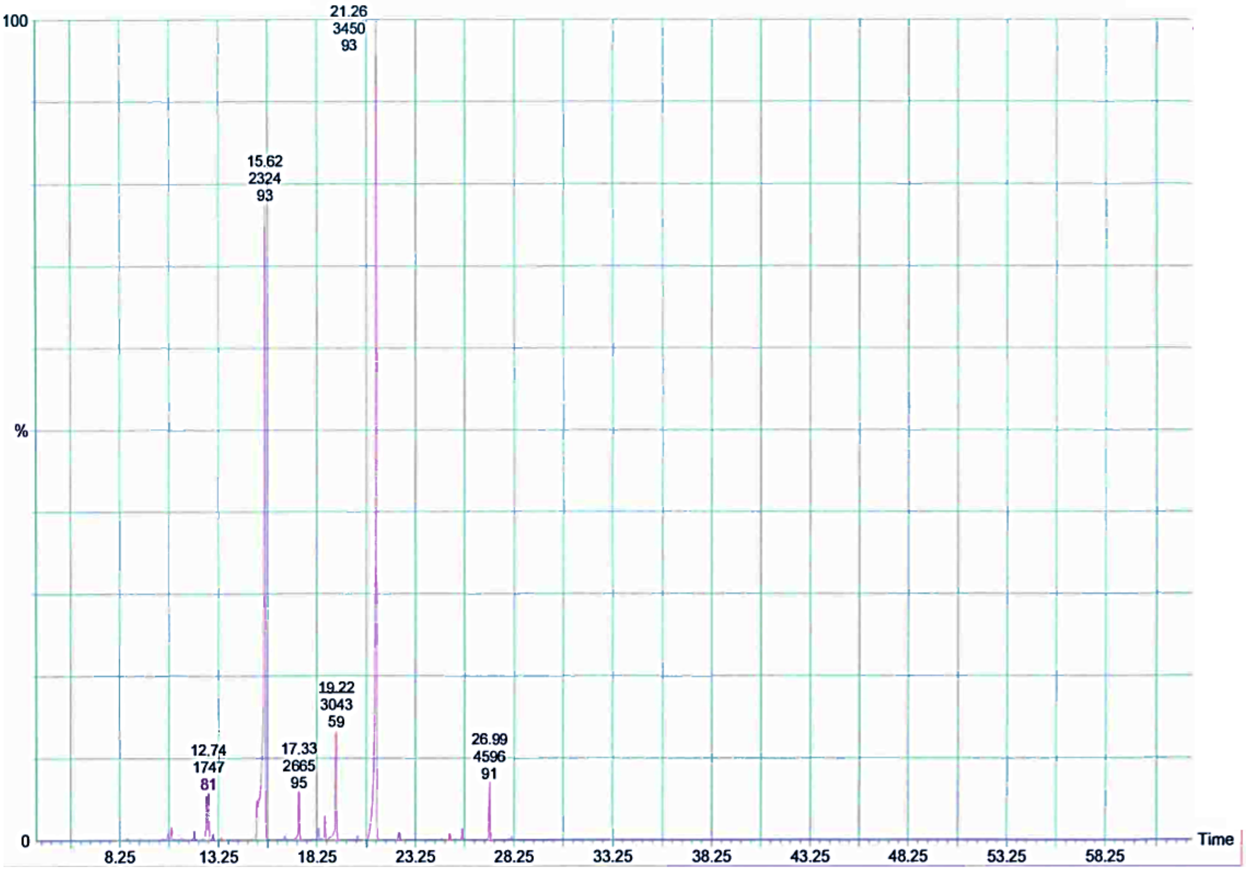

Figure S2. GC/MS chromatogram of *O. syriacum* essential oil

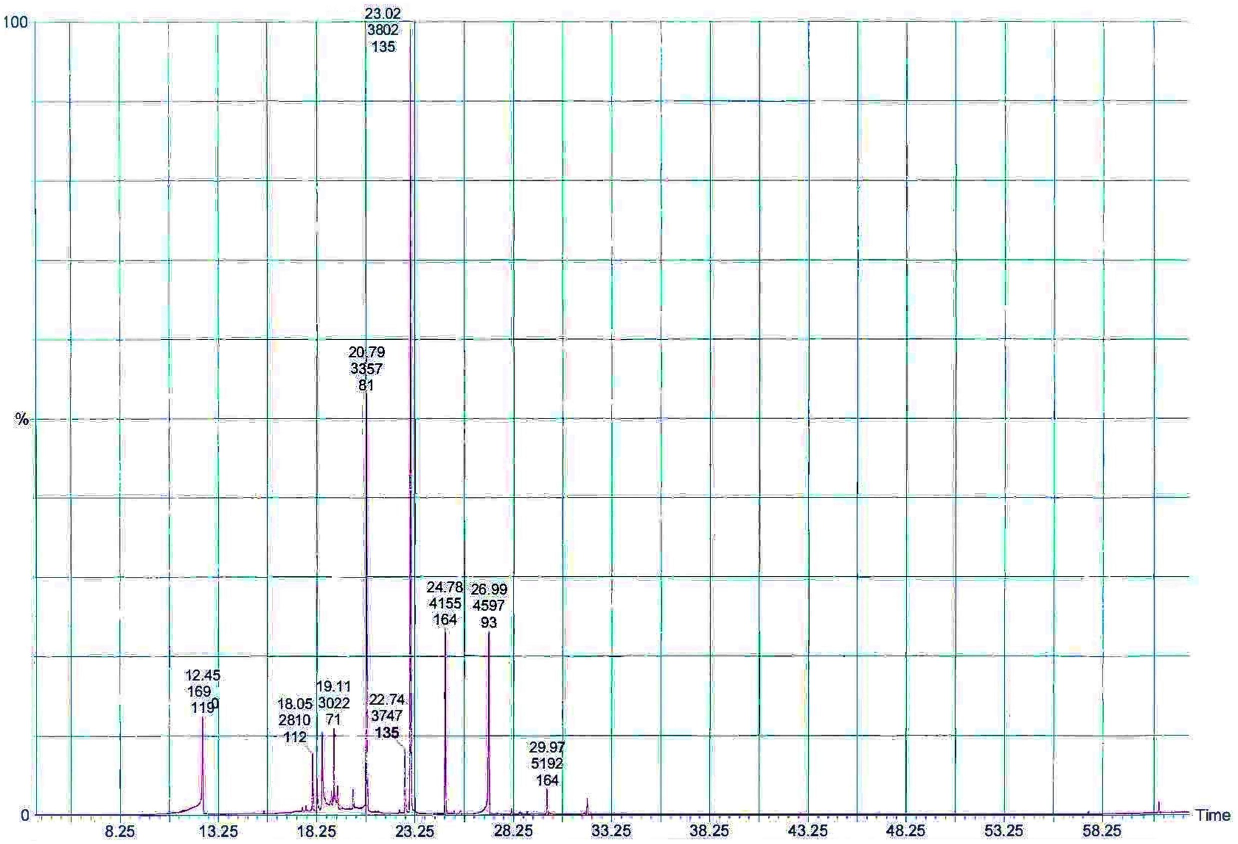

Table S1. Data analysis for the whole cell recordings.

	**GluA1**	**n**	**Plus thyme oil**	**n**	**Plus Lavendar**	**n**
Amplitude (pA)	724±101	16	710±74^{ns}	20	732±186^{ns}	20
τω deact (ms)	2.4±0.1	12	4.5±0.2***	12	7.2±0.9	12
τω des (ms)	3.2±0.1	16	5.6±0.7***	20	9.2±0.9	20
	**GluA1/2**	**n**	**Plus thyme oil**	**n**	**Plus Lavendar**	**n**
Amplitude (pA)	278±48	12	280±73^{ns}	12	207±40^{ns}	20
τω deact (ms)	2.3±0.1	12	4.8±0.5***	12	8.1±0.8***	10
τω des (ms)	5.6±0.6	12	5.3±0.5^{ns}	12	28.8±3.2***	20
	**GluA2**	**n**	**Plus thyme oil**	**n**	**Plus Lavendar**	**n**
Amplitude (pA)	935±67	15	920±56^{ns}	20	955±143^{ns}	20
τω deact (ms)	2.2±0.1	12	3.8±0.6***	12	5.6±0.3***	12
τω des (ms)	2.5±0.1	15	4.7±0.5***	20	8.4±0.9***	20

Significance compared with AMPAR expressed alone (*); p-value (one-way ANOVA): * < 0.05, ** < 0.01, ***< 0.001, ns – not significant.
